# Exosomes from *Plasmodium*-infected hosts inhibit tumor angiogenesis in a murine Lewis lung cancer model

**DOI:** 10.1038/oncsis.2017.52

**Published:** 2017-06-26

**Authors:** Y Yang, Q Liu, J Lu, D Adah, S Yu, S Zhao, Y Yao, L Qin, L Qin, X Chen

**Affiliations:** 1 State Key Laboratory of Respiratory Disease, Guangzhou Institutes of Biomedicine and Health, Chinese Academy of Sciences, Guangzhou, Guangdong, PR China; 2 University of Chinese Academy of Sciences, Beijing, PR China

## Abstract

Previous research to investigate the interaction between malaria infection and tumor progression has revealed that malaria infection can potentiate host immune response against tumor in tumor-bearing mice. Exosomes may play key roles in disseminating pathogenic host-derived molecules during infection because several studies have shown the involvement and roles of extracellular vesicles in cell–cell communication. However, the role of exosomes generated during *Plasmodium* infection in tumor growth, progression and angiogenesis has not been studied either in animals or in the clinics. To test this hypothesis, we designed an animal model to generate and isolate exosomes from mice which were subsequently used to treat the tumor. Intra-tumor injection of exosomes derived from the plasma of *Plasmodium*-infected mice provided significantly reduced Lewis lung cancer growth in mice. We further co-cultured the isolated exosomes with endothelial cells and observed significantly reduced expression of VEGFR2 and migration in the endothelial cells. Interestingly, high level of micro-RNA (miRNA) 16/322/497/17 was detected in the exosomes derived from the plasma of mice infected with *Plasmodium* compared with those from control mice. We observed that overexpression of the miRNA 16/322/497/17 in endothelial cell corresponded with decreased expression of VEGFR2, inhibition of angiogenesis and inhibition of the miRNA 16/322/497/17 significantly alleviated these effects. These data provide novel scientific evidence of the interaction between *Plasmodium* infection and lung cancer growth and angiogenesis.

## Introduction

Angiogenesis is vital for tumor growth, survival and progression.^1, 2, 3^ Vascular endothelial growth factor (VEGF) is one of the key angiogenic factors that drive vascular growth by attracting and activating cells from within the microenvironment of the tumor.^4^ Vascular endothelial cell surface contains VEGF binding sites which signal via three receptor tyrosine kinases (VEGFR1, 2 and 3) and are regulated at multiple levels. VEGFR2 is the major regulator of the angiogenic effect of VEGF.^5, 6^ The signaling cascades of VEGF regulate vascular permeability modulation, extracellular matrix degradation, and cell migration, proliferation, and survival. Multiple downstream signaling pathways depend on VEGF-VEGFR2 binding, including the PLC (phosphoinositide phospholipase C)-γ pathway in controlling cell proliferation and vascular permeability, the FAK (focal adhesion kinase)/paxillin pathway in regulating cytoskeletal rearrangement and cell migration, the Ras/MAPK (mitogen-activated protein kinase) pathway in regulating gene expression and cell proliferation, and the PI3K (phosphatidylinositide 3-kinases)/AKT (also known as Protein kinase B (PKB)) pathway in regulating cell survival.^7, 8^

Exosomes are 30–100 nm lipid bilayer membrane vesicles that contain various types of macromolecules, including nucleic acids, carbohydrates, proteins and lipids. More recent studies have identified that exosomes are rich in mRNA, micro-RNA (miRNA or miR) and other non-coding RNAs.^9, 10^ Previous studies have reported that exosomes are secreted by numerous cell types, including immune cells, cancer cells, stem cells, and neurons.^11^ Furthermore, exosomes produced during an infection can be either pathogen or host derived. Pathogens such as helminths, fungi, bacteria and parasitic protozoa, including species of *Trypanosoma*, *Trichomonas, Toxoplasma*, *Leishmania* and *Plasmodium*, also secrete exosomes or extracellular vesicles (EVs).^12, 13^ Some studies have found that injection of exosomes derived from dendritic cells subjected to antigens of the obligate intracellular parasite *Toxoplasma gondii in vitro* induced an immune response that conferred protection against pathogen infection.^14^

Malaria, which is caused by an intracellular parasite from the *Plasmodium* genus, is the most common parasitic infection in humans. In recent studies, plasma red blood cell-derived microparticle (MP) levels were elevated in patients with *Plasmodium falciparum* (*P. falciparum*) in proportion to disease severity and were also elevated in patients with *Plasmodium vivax* and *Plasmodium malariae*.^15, 16^ Studies have demonstrated that exosomes isolated from *Plasmodium yoelii*-infected reticulocyte cultures carry antigens and are involved in immune modulation.^17^ Our previous study indicated that *P. yoelii* 17XNL infection significantly suppressed Lewis lung cancer (LLC) cell growth through induction of innate and adaptive antitumor responses in a murine model. Furthermore, we found that *Plasmodium* infection inhibited tumor angiogenesis;^18^ however, the underlying mechanisms are not well understood. Therefore, we hypothesized that exosomes derived from *Plasmodium*-infected mice inhibit tumor angiogenesis. Here, we observed the functions of exosomes derived from the plasma of *Plasmodium*-infected mice in angiogenesis. More importantly, we identified that specific miRNAs overexpressed in these exosomes have anti-angiogenic functions. Our studies provide a novel understanding of the interaction between *Plasmodium* infection and lung cancer.

## Results

### *Plasmodium*-infected animals exhibited increased exosome secretion

**Figure 1:**
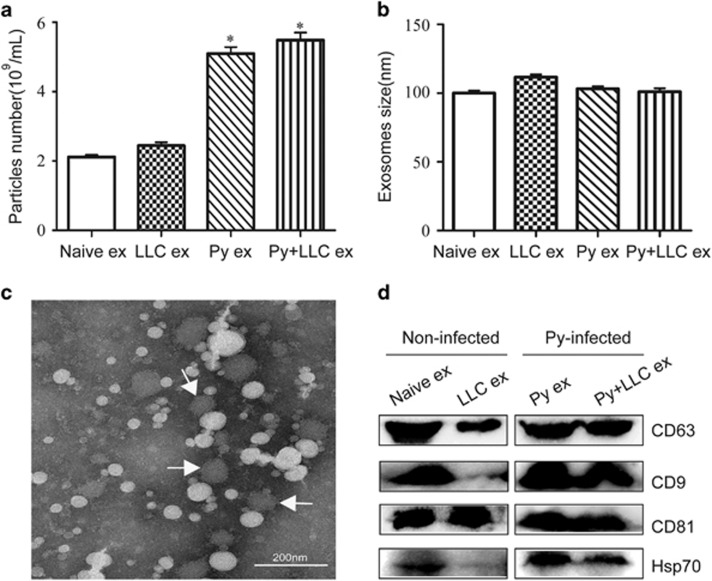
Identification of exosomes characteristics. (**a**) Using Nanosight to detect particle numbers of exosomes isolated from plasma of mice in different groups (*P<0.05, compared with naïve ex and LLC ex groups). (**b**) Using Nanosight to detect size of exosome isolated from plasma of mice in different groups. (**c**) A representative electron microscopic image of exosomes from mice plasma; scale bar 200 nm. (**d**) Confirmed by western blot for CD63, CD9, CD81 and Hsp70 exosomes markers from plasma of mice.

To detect exosome secretion in the different groups, exosomes were analyzed for particle numbers, size and morphology distribution using Nanosight system and TEM (transmission electron microscope), respectively. The results indicated that the mice infected with Py secreted higher levels of exosomes in plasma. Compared with the Py groups, the LLC and naïve groups had low exosomal particle numbers (Figure 1a). The exosomes from all of the groups were homogeneous in size (median diameter, 100 nm) and similar in morphology (Figures 1b and c). We further confirmed the presence of the known exosome markers CD63, CD9, CD81and Hsp70 by Western blot analysis (Figure 1d).

### Exosomes isolated from *Plasmodium*-infected mice inhibited tumor growth and angiogenesis

**Figure 2:**
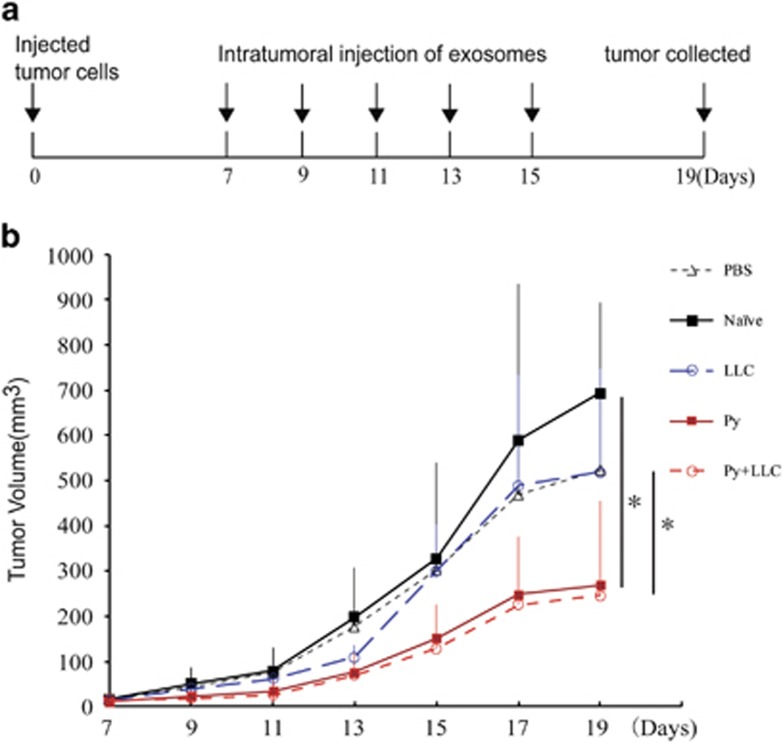
Exosomes suppressed tumor growth. (**a**) Longitudinal exosomes intra-tumor injection (each groups *n*=6). (**b**) C57BL/6 mice were inoculated with 5 × 10^5^ lewis lung cells subcutaneously. Exosomes were injected intra-tumor from day 7 when tumor reached 3 × 3 mm^2^ in size. Tumor growth was monitored (*V*=*ab*^2^/*2*). Bars correspond to mean±s.d. (*P<0.05 Naïve ex versus Py ex; LLC ex versus Py+LLC ex).

**Figure 3:**
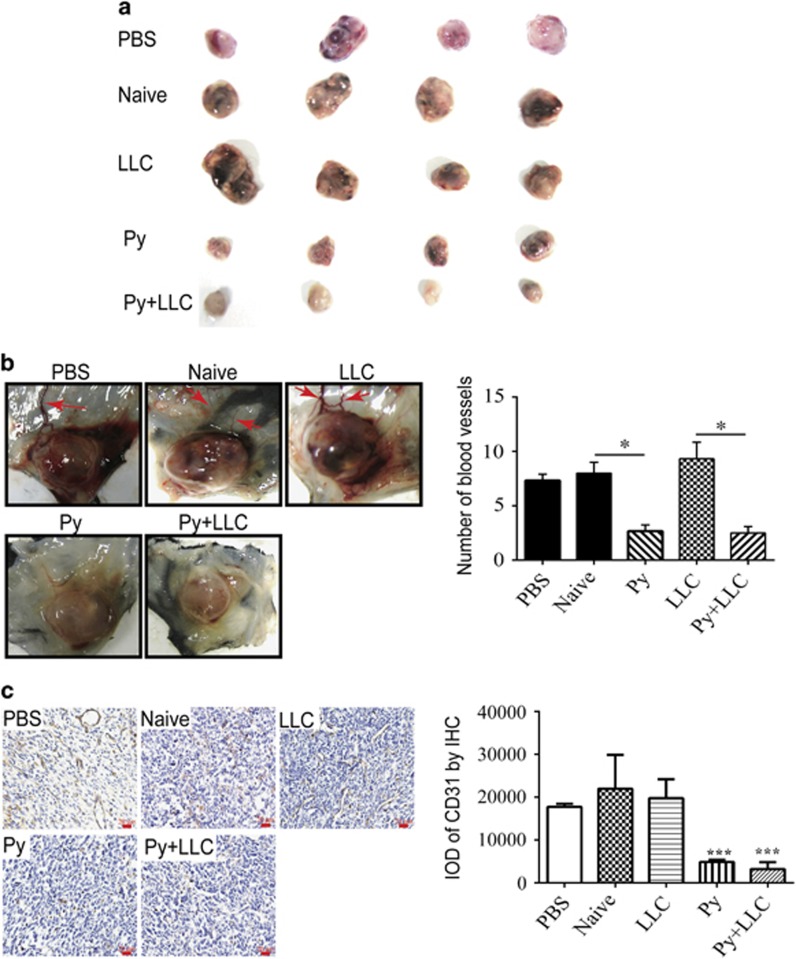
Exosomes inhibited angiogenesis. (**a**) At 19 days, tumors were collected and presented as shown. (**b**) Tumors displayed varying degrees of feeding tumor vessels along the undersurface of the surrounding dermis at 19 days post inoculation. Quantification of blood vessels feeding to tumors treated with exosomes of different groups, or PBS. *n*=5. (*P<0.05). (**c**) Immunohistochemical analysis of CD31 expression in tumor tissues. Scale bar 20 μm (***P<0.001 Py ex and Py+LLC ex groups compared with PBS, Naïve ex and LLC ex groups).

To investigate the effect of exosomes from the plasma of mice infected with *P. yoelii* on tumor growth, we established an LLC mouse model. When the tumor volume reached 3 × 3 mm^2^ (7 days), 50 μg of exosomes (ex) from different groups were injected into each mouse via intra-tumor injection once every other day for 10 days (Figure 2a). During the period of treatment, tumor growth was significantly suppressed in the Py ex and Py+LLC ex groups compared to the naïve ex and LLC ex groups (*P*<0.05) (Figure 2b). On day 19, we collected the tumors and found that the phosphate-buffered saline (PBS), naïve ex and LLC ex treatments had tumors with numerous large feeding blood vessels; however, the blood vessels from adjacent dermis that feeds the tumor with nutrients and metabolites were significantly reduced in tumors from Py ex and Py+LLC ex treatment groups (Figures 3a and b). To further confirm the effect of plasma exosomes from *Plasmodium*-infected mice on angiogenesis, the exosomes from different groups were injected intra-tumorally as previously described. Tumors were collected 19 days post inoculation. Hematoxylin and eosin staining showed that the number of microvessels in the Py exosome and Py+LLC exosome treatment groups was markedly less than that in the PBS, naïve exosome and LLC exosome groups (Supplementary Figure S1). We used the exosomes marker CD63 antibody and red blood cell marker CD235a antibody to validate the exosomes in tumor tissue by immunohistochemistry (IHC). The IHC revealed that exosomes could be taken up by cells *in vivo* (Supplementary Figure S2). In the endothelial cells, angiogenesis marker, CD31, can be used to show the extent of tumor angiogenesis and imply a rapidly growing tumor.^19^ IHC analysis revealed that CD31 expression in the PBS, naïve exosome and LLC exosome treatment groups was higher than in the *Plasmodium*-infected exosome treatment groups (Figure 3c). Taken together, these results suggest that exosomes derived from malaria-infected mice can inhibit angiogenesis and suppress LLC growth *in vivo*.

### Exosomes could be taken up by endothelial cells and at least a part of exosomes derived from red blood cells

**Figure 4:**
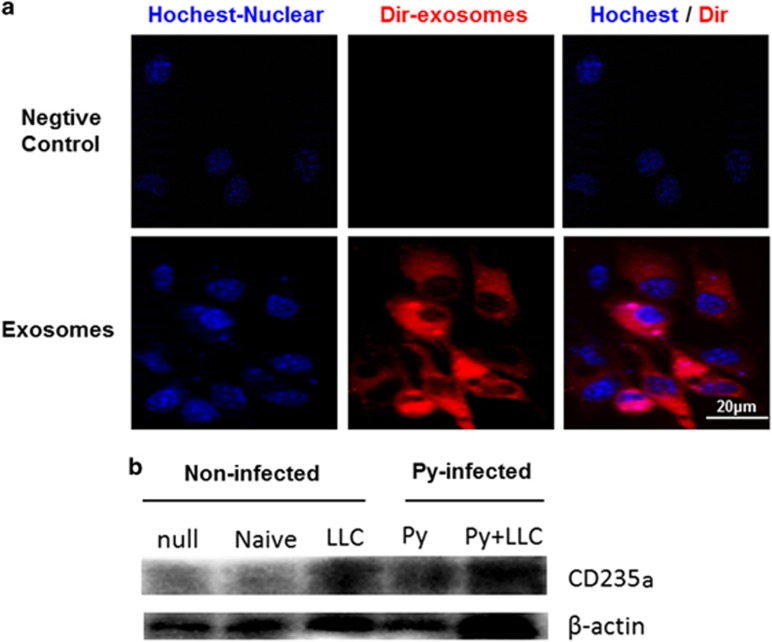
Exosomes uptake by MS1 cells *in vitro*. (**a**) Confocal images of cultures exposed to exosomes at 6 h. The red channel is representative of Dir emission, the blue channel is Hoechst nuclear stain. Fluorescent signals are merged with transmission images. Scale bar is 20 μm. (**b**) MS1 co-culture with different exosomes at 24 h and uptake confirmed by western blot for red blood cell special marker CD235a.

We labeled exosomes with the lipohilic dye Dir and exposed them to MS1 cell cultures for 6 h. Confocal images on live cell confirmed that the exosomes were well visible (Figure 4a). Some researchers have used red blood cell marker CD235a to identify the red blood cell-derived MPs in malaria patients.^15^ To determine whether some exosomes were derived from the red blood cells and taken up by MS1 cells, we co-cultured different groups’ exosomes with MS1 cells for 24 h. Then we detected CD235a in the MS1 cell lysate using western blot assay. The results showed that the CD235a could be detected in the lysate of the LLC, Py and Py+LLC exosomes’ groups (Figure 4b). It is suggested that exosomes from plasma of Py-infected mice include those derived from red blood cells.^15^ Our current results suggested that the uptake of exosomes by endothelial cells did occur and some of the exosomes were derived from the red blood cells.

### Exosomes derived from *Plasmodium*-infected host plasma inhibited tube formation and migration of endothelial cells

**Figure 5:**
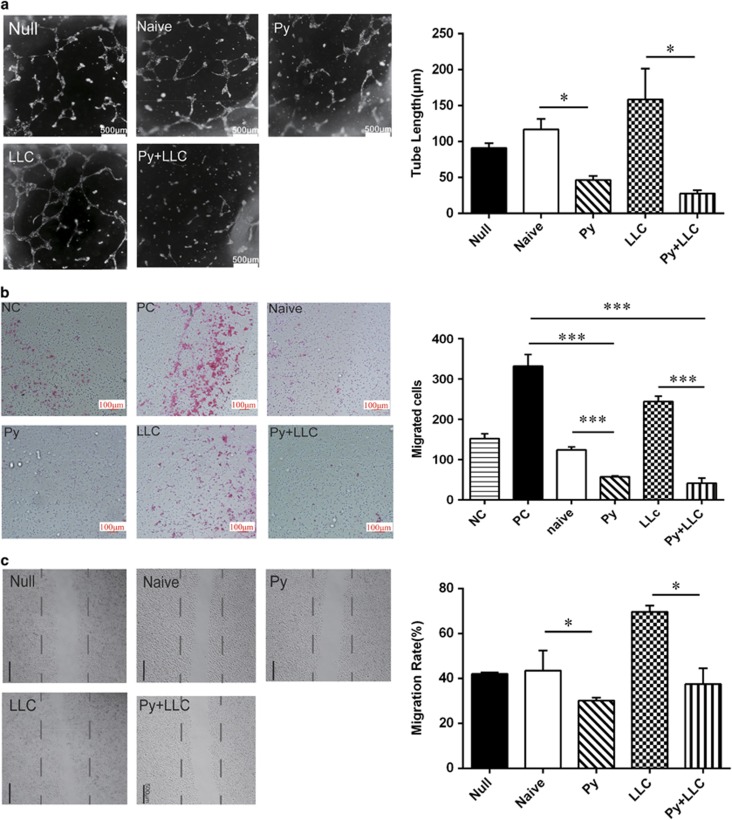
The effect of exosomes on tube formation and migration in endothelial cell. (**a**) Endothelial cell tube formation assay showed interference of network assembly of MS1 cells on pre-solidified Matrigel in medium containing exosomes. Scale bar 500 μm (*P<0.05). (**b**) The invasion activity of MS1 cell incubated with different exosomes using transwell method. Scale bar 100 μm (NC: Negative control, PC: Positive control, ***P<0.001). (**c**) Endothelial cell migration was measured after co-culture with different groups exosomes for 24 h. Lines indicated the edge of the ‘wound’ directly after making the scratch. Statistical analysis of the width of the scratches is shown on the right (*P<0.05).

MS1 cells (an endothelial cell line) co-cultured with exosomes from the plasma of Py-infected mice and non-infected mice were sequenced using next generation sequencing technology. The RNA-Seq results highlighted the occurrence of angiogenesis and endothelial cell migration based on the expression changes in a large number of mRNAs (Table 1). To confirm the sequencing results, we used a tube formation assay to test the effect of exosomes on angiogenesis *in vitro*. The Py exosomes and Py+LLC exosomes inhibited the formation of a tube-like network of MS1 cells on Matrigel pre-coated plates, whereas tube formation was increased significantly in MS1 cells incubated with naïve exosomes and LLC exosomes (Figure 5a). We performed transwell migration assays and found that co-culturing MS1 cells with LLC cells markedly increased the migration of MS1 cells. However, MS1 cells co-cultured with exosomes from *Plasmodium*-infected mouse plasma showed significantly decreased migration (Figure 5b). Similarly, MS1 cell motility was significantly decreased by plasma exosomes from *Plasmodium*-infected mice in a scratch wound assay compared with that of the naïve and LLC exosome groups (Figure 5c). The above data suggested that plasma exosomes from malaria-infected mice were capable of impeding endothelial cell migration, leading to decreased tumor angiogenesis. Additional information on the effect of exosomes on endothelial cell proliferation is available in Supplementary Figure S6.

### Plasma exosomes from malaria-infected mice downregulated VEGFR2 expression in endothelial cells

**Figure 6:**
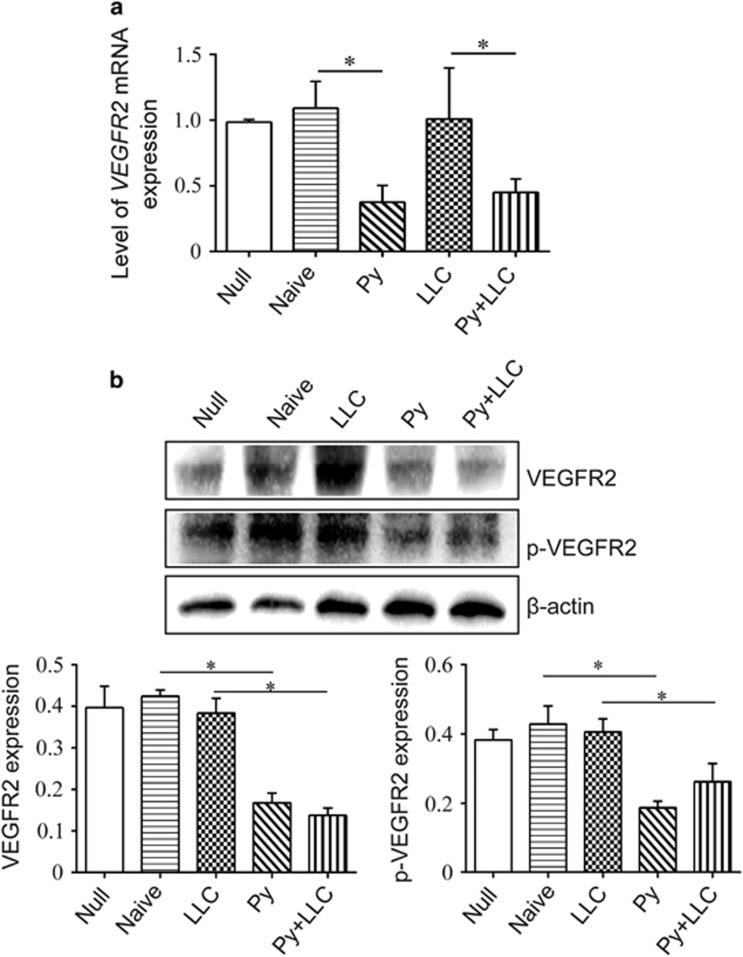
Exosomes inhibited vegfR2 expression in MS1 cells. (**a**) MS1 cells were cultured *in vitro* and exosomes were added in the culture medium for 24 h. VEGFR2 mRNA expression was detected using qPCR (*P<0.05). (**b**) Western blotting analysis of phospho-VEGFR2 and VEGFR2 expression in MS1 cells co-culture with exosomes; Quantitative results of western blotting assay showed thatVEGFR2 and phospho-VEGFR2 expression (*P<0.05).

VEGF receptor 2 is the major VEGF signaling receptor that regulate sprouting angiogenesis.^20^ We hypothesized that plasma exosomes from malaria-infected mice inhibited angiogenesis through decreasing VEGFR2 expression in endothelial cells. To validate this hypothesis, exosomes (ex) were added to the culture medium of MS1 cells. After a 24-h treatment, we found that VEGFR2 and phospho-VEGFR2 were remarkably decreased in the Py ex and Py+LLC ex groups (Figures 6a and b). In addition, we also detected the VEGFR2 downstream signal pathway (Supplementary Figure S3a). Unfortunately, proteins in the MAPK pathway and Src/Akt pathway did not significantly differ among the different groups (Supplementary Figure S3b). Ki8751 potently and selectively inhibits VEGFR2^21^ so that we used it as control. It is known that cytoskeletal rearrangement and cell migration depend on the FAK pathway. Western blot results demonstrated that FAK was decreased in the Py ex and Py+LLC ex groups. The same result was also obtained from cells treated with a VEGFR2 kinase inhibitor Ki8751 at 50 nM (Supplementary Figure S3b). Delta-like 4 (DLL4), a membrane-bound ligand belonging to the Notch signaling family, plays a key role in vascular development and angiogenesis. We also found that plasma exosomes from malaria-infected mice reduced DLL4 expression in ECs (Supplementary Figure S3c). Furthermore, Ki8751 effectively decreases MS1 tube formation (Supplementary Figure S3d), which has the same effect of exosomes from Py-infected on MS1 cells (Figure 4a). These results suggested that the effects of the exosomes from Py-infected hosts plasma on the VEGF-α/VEGFR2 pathway result in decreased expression of not only VEGFR2 but also FAK and DLL4, which are important in vascular development.

### miRNAs overexpressed in plasma exosomes from *Plasmodium*-infected mice targeted VEGFR2

**Figure 7:**
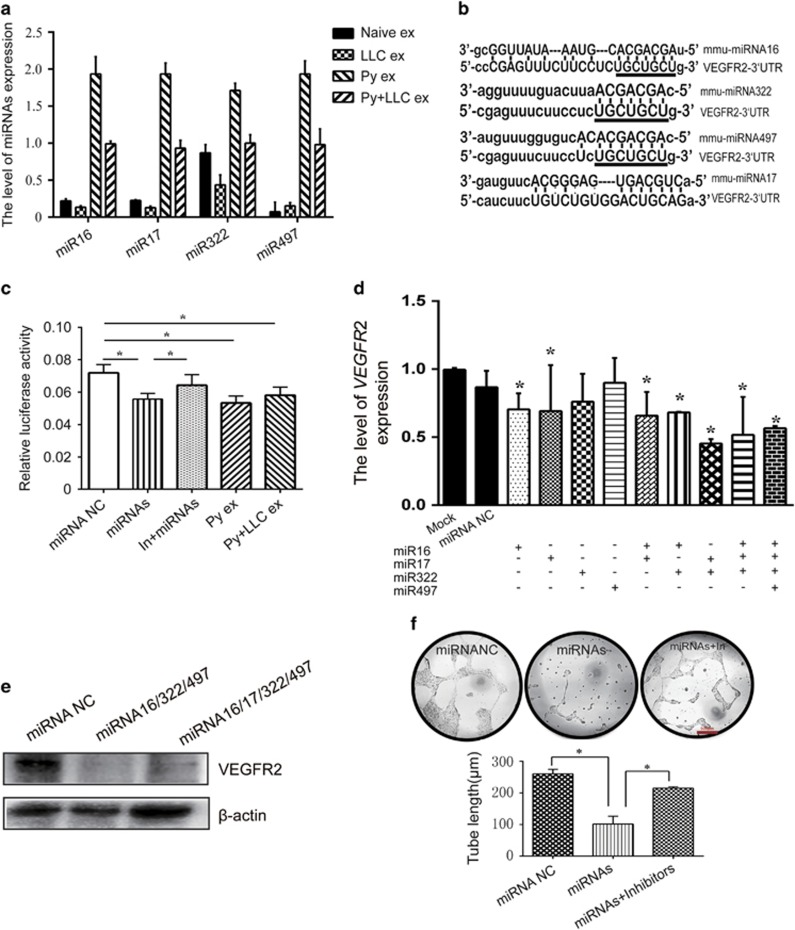
miRNAs is overexpressed in plasmodium-infected mice plasma exosomes, downregulated VEGFR-2 and inhibited tube formation. (**a**) qPCR detected the level of miRNAs expression in exosomes of four groups. (**b**) VEGFR2 is a target gene of miR(16-5p/322-5p/497-5p/17-5p). (**c**) Luciferase reporter assay was performed using 293T cells as described in the Materials and methods section (*P<0.05,compared with miRNA negative control; # *P*<0.05, compared with miRNAs add inhibitors). (**d**) The relative expression of VEGFR2 mRNA in MS1 cells transfected with different combination of miRNAs based on qPCR (*P<0.05, compared with miRNA negative control). (**e**) The protein level of VEGFR2 in MS1 cells transfected with two combination of miRNAs based on western blotting. (**f**) Endothelial cell tube formation assay showed interference of network assembly of MS1 cells on pre-solidified Matrigel. Scale bar 500 μm (miRNA NC: Transfection of miRNA negative control; miRNAs: Transfection of miR (16-5p/322-5p/497-5p/); miRNAs+inhibitors: Transfection of miR (16-5p/322-5p/497-5p/17-5p) and inhibitors. *P<0.05).

qRT–PCR analysis showed twofold overexpression of miRNAs (16-5p/17-5p/322-5p/497-5p) in Py exosomes compared with naïve exosomes and LLC exosomes. Consistently, the levels of Py+LLC exosomal miRNAs (16-5p/17-5p/322-5p/497-5p) were 1.5-fold higher than those of the two control groups (Figure 7a). Bioinformatics analysis showed predicted binding sites between these miRNAs (16-5p/17-5p/322-5p/497-5p) and VEGFR2. Interestingly, the miRNA 16-5p/322-5p/497-5p was predicted to target the same site in VEGFR2. miR-17-5p targeted another site in VEGFR2 (Figure 7b). Therefore, we conducted a dual-luciferase reporter assay to determine whether these miRNAs (16-5p/17-5p/322-5p/497-5p) could directly bind to the 3′-UTRs of VEGFR2. Transient transfection of a VEGFR2-luc reporter and miRNA (16-5p/17-5p/322-5p/497-5p) mimics into 293T cells significantly reduced luciferase activity compared with the control mimics. Interestingly, Py and Py+LLC exosomes could reduce luciferase activity significantly (Figure 7c). Therefore, the results suggested that *Plasmodium* infection upregulated the levels of miRNA (16-5p/17-5p/322-5p/497-5p) expression in the plasma exosomes from the host. These exosomes probably inhibited angiogenesis via miRNA upregulation to suppress VEGFR2 expression.

### miRNAs (16-5p/17-5p/322-5p/497-5p) inhibited VEGFR2 expression in and tube formation by endothelial cells

To confirm the effect of each miRNA identified in the exosomes on VEGFR2, various miRNA mimics were transfected into MS1 cells. Using qPCR, we found that miR16-5p and miR17-5p significantly inhibited VEGFR2 mRNA expression but that miR322-5p and miR497-5p had no significant effect. In addition, we transfected different miRNA combinations into MS1 cells and found that the miRNA 16-5p/322-5p/497-5p decreased VEGFR2 mRNA expression remarkably. Consistently, the miRNA 16-5p/322-5p/497-5p and miR17-5p transfected together into MS1 had the same effect (Figure 7d). Our western blot assay also showed that the miRNA 16-5p/322-5p/497-5p and 16-5p/17-5p/322-5p/497-5p suppressed VEGFR2 expression (Figure 7e).

To determine whether the miRNA 16-5p/17-5p/322-5p/497-5p could suppress tube formation by MS1 cells, we conducted a tube formation experiment. Using a tube formation assay to test the effect of miRNAs on angiogenesis *in vitro*, we found that the miRNA 16-5p/17-5p/322-5p/497-5p could inhibit the formation of a tube-like network by MS1 cells on Matrigel pre-coated plates, whereas MS1 cells incubated with miRNA inhibitors showed increased tube formation (Figure 7f). The above data suggested that the miRNA 16-5p/17-5p/322-5p/497-5p decreased VEGFR2 expression and inhibited EC tube formation. The effects of the miRNAs were similar to those of the exosomes from Py-infected host plasma.

### Exosomes from Py-infected host plasma inhibited VEGFR2 expression and tube formation via the miRNA 16-5p/17-5p/322-5p/497-5p

**Figure 8:**
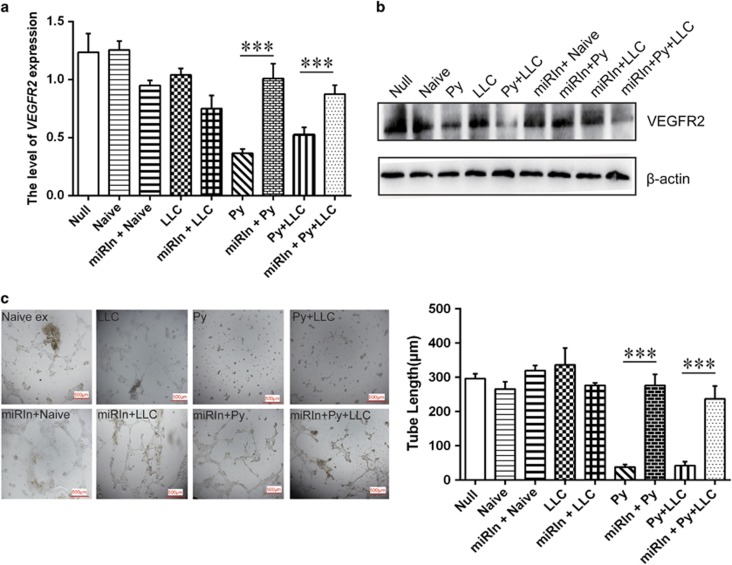
miRNAs inhibitors rescued the effect of exosomes inhibition on VEGFR2 expression and tube formation in endothelial cells. (**a**) The relative expression of VEGFR2 mRNA in MS1 cells co-cultured with different groups exosomes and transfected with miRNAs inhibitors based on qPCR (***P<0.01). (**b**) The protein level of VEGFR2 in MS1 cells co-culture with Py or Py+LLC exosomes and miRNAs inhibitors based on western blotting. (**c**) Endothelial cell tube formation assay showed interference of network assembly of MS1 cells on presolidified Matrigel. Scale bar 500 μm (miRIn: Transfection of miR (16-5p/322-5p/497-5p/17-5p) inhibitors. ***P<0.01).

Although we found that the miRNA 16-5p/322-5p/497-5p and miRNA 17-5p were overexpressed in exosomes derived from *Plasmodium*-infected mouse plasma, the effects of these exosomal miRNAs on angiogenesis were still unknown. To understand whether the miRNA 16-5p/17-5p/322-5p/497-5p in Py and Py+LLC exosomes plays an important role in anti-angiogenesis, miRNA (16-5p/17-5p/322-5p/497-5p) inhibitors were transfected into MS1 cells and co-cultured with the exosomes for 24 h. Using qPCR and western blot, we found that miRNA (16-5p/17-5p/322-5p/497-5p) inhibitors could attenuate the inhibition of VEGFR2 expression significantly (Figures 8a and b). Using the tube formation assay to confirm this effect, we found that when co-cultured with exosomes from Py-infected host plasma, the miRNA inhibitors remarkably increased tube-like network formation of MS1 cells on Matrigel pre-coated plates (Figure 8c), suggesting that the Py and Py+LLC exosomes inhibited VEGFR2 expression and tube formation through the miRNA 16-5p/17-5p/322-5p/497-5p.

**Figure 9:**
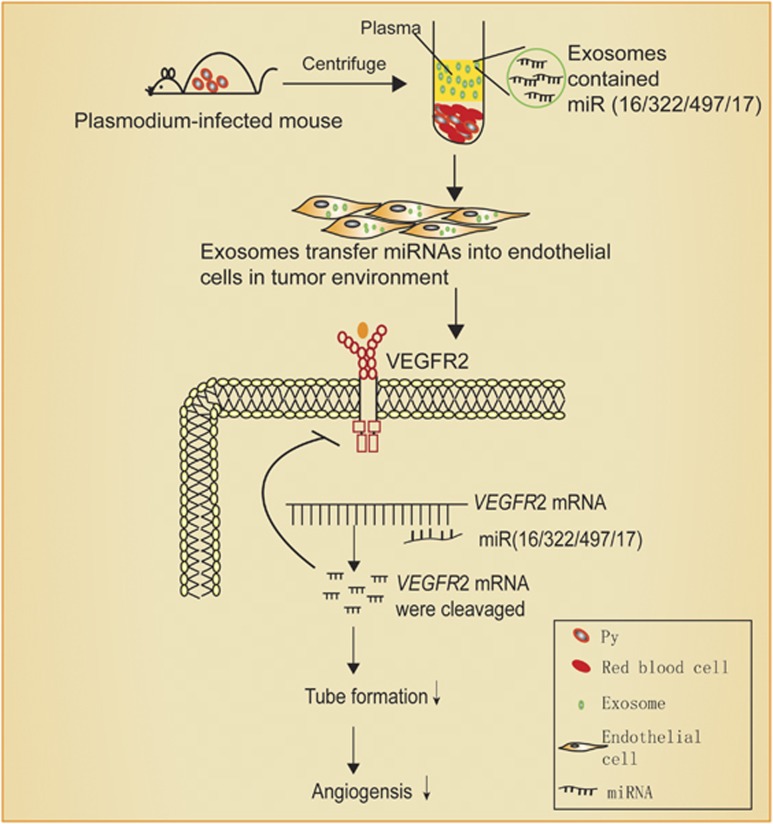
Schematic of plasma exosomes from plasmodium-infected mice inhibition of angiogenesis through miR(16/322/497/17) to target VEGFR2.

In the tumor microenvironment, the exosomes from *Plasmodium*-infected host plasma were taken up by vascular endothelial cells. The miRNA 16-5p/17-5p/322-5p/497-5p in the exosomes could bind to and degrade VEGFR2 mRNA in endothelial cells, thereby inhibiting angiogenesis (Figure 9).

## Discussion

Clinical studies have highlighted elevated plasma red blood cell-derived MP concentrations in patients with *P. falciparum* malaria in relation to disease severity. Other reports have stated that these MP concentrations were also elevated in patients with *P. vivax* and *P. malariae* infections. The release of EVs may be due to endothelial activation or a direct mechanical result of the cytoadherence of infected red blood cells to the endothelium.^15, 16, 22, 23, 24^ Consistently, in a murine model, our studies found that the plasma exosome concentration in *P. yoelii*-infected mice was approximately threefold higher than in naïve mice and tumor-bearing mice. The diameters of the exosomes were approximately 100 nm as demonstrated by transmission electron microscopy.

Pathogens, parasitic protozoa, helminths (flat- and round-worms), fungi and bacteria also secrete EVs.^25, 26, 27, 28^ Cells infected with pathogens shed EVs that bear antigens specific to the pathogens. Reticulocytes infected with Py are known to shed exosomes that carry antigens which protected mice from lethal infections.^17^ Pierre-Yves and colleagues found that microvesicles derived from *P. falciparum*-infected red blood cells RMV (microvesicles derived from P. falciparum-infected red blood cells) are mainly released during the asexual parasite cycle prior to parasite egress. RMVs have been demonstrated to exert potent immunomodulatory effects on human primary macrophages and neutrophils.^29^ In addition, cellular activities can be regulated when exosomes are taken up by acceptor cells. Previous studies demonstrated that MPs derived from tumor cell serves as carriers to deliver therapeutic agents to tumor cells, for effective tumor cell killing with reduced adverse effects.^30^ Intra-tumoral injection of proteins, viral vectors and cells has been used in tumor treatment. Studies have shown that intra-tumoral injection of MSCs-derived exosomes reduced the growth of glioma xenograft in a rat brain tumor model.^31, 32^ Our present *in vivo* experiment indicated that exosomes from malaria-infected mice directly injected into tumor tissues suppressed LLC growth and inhibited the formation of intradermal blood vessels that supply tumor tissues. These results suggested that exosomes from Py-infected mice inhibit tumor-induced neovascularization and endothelial cell migration.

Tumor growth and development require a supply of nutrients and oxygen. Tumor-associated neovasculature generated by the process of angiogenesis addresses these needs. The major mediators of tumor angiogenesis are VEGF-A and VEGF signals, which are regulated primarily through VEGFR2, which is expressed at increased levels by endothelial cells involved in angiogenesis.^1, 20^ The mice bearing a mutant VEGF gene only in vascular endothelial cells suggest that very low levels of autocrine-acting VEGF mediate endothelial cell survival and vascular homeostasis by signaling through intracellular VEGFR2.^33^ Our *in vitro* experiments showed that exosomes from the plasma of Py-infected mice suppressed VEGFR2 expression by endothelial cells. Consistently, the *in vivo* experiments showed the same results. Therefore, Py-infected mice secreted exosomes that inhibited angiogenesis via downregulation of VEGFR2 expression. In addition, the transwell assay and wound-healing assay results revealed that Py exosomes and Py+LLC exosomes decreased endothelial cell migration. Interestingly, either LLC co-culturing with endothelial cells or LLC exosome supplementation in the culture medium significantly increased endothelial cell migration, which further highlighted the effect of exosomes derived from *Plasmodium*-infected mice. Furthermore, the tube formation assay confirmed that exosomes derived from the plasma of *Plasmodium*-infected hosts significantly decreased endothelial cell tube formation. Collectively, these results indicated that exosomes from malaria-infected mice suppressed angiogenesis through inhibition of both VEGFR2 expression and endothelial cell migration.

EVs naturally carries RNA molecules which when delivered to recipient cells can elicit functional changes. Several studies have confirmed the ability of exosomes to transfer miRNA into recipient cells.^9, 34, 35, 36^ These findings indicate that EVs use natural mechanisms for the transfer and internalization of cellular components and highlight a potential role for EVs in small RNA delivery. Interestingly, *P. falciparum*-infected red blood cells are known to deliver genes between parasites through exosome-like vescicles communication.^37^ Interestingly, previous study found that *P. falciparum*-infected red blood cells (iRBC)-derived EVs possess miRNAs that can regulate target genes in recipient cells. Several miRNA species in EVs bound to Ago2 and form functional complexes. Moreover, researchers found the transfer of iRBC-derived EVs into endothelial cells, repression of miRNA target genes and alteration of endothelial barrier properties.^38^ We used qPCR to confirm that the miRNA 16-5p/17-5p/322-5p/497-5p was highly overexpressed in the Py and Py+LLC exosome groups. However, the Py+LLC exosomes expressed lower levels of the four miRNAs compared to the Py exosomes. The reason for this phenomenon is unclear.

Furthermore, miRNAs have been found to modulate the expression of genes involved in biological processes. The progression of the cell cycle from the G_0_ to S phase is believed to be mediated by some members of the miR-16 family, thereby inducing cell-cycle arrest and acting as potent tumor suppressors.^39, 40, 41^ Overexpression of either miR-15b or miR-16 repressed the expression of the target protein VEGF and the proliferation of early EPC (endothelial progenitor cells), while the opposite phenomenon was observed upon knockdown of these miRNAs. Increasing miR-16 expression might be a promising strategy for tumor therapy by repressing tumor angiogenesis and inducing tumor cell death through targeting VEGF and BCL2.^42, 43^ miR-17 is negative regulator of angiogenesis in ECs *in vitro* and *in vivo*. Overexpression of miR-17 inhibited EC sprouting in a three-dimensional spheroid model, while inhibition of this miRNA enhanced spheroid sprouting.^44, 45^ Our *in vitro* studies demonstrated that the miRNA 16-5p/17-5p/322-5p/497-5p suppressed VEGFR2 expression in MS1 cells. In addition, transfection of miRNA (16-5p/17-5p/322-5p/497-5p) inhibitors in conjunction with either Py or Py+LLC exosomes attenuated the inhibitory effect. Using bioinformatics, we analyzed the target site of these miRNAs and found that these miRNAs (16-5p/322-5p/497-5p) share the same target site in the 3′-UTR of VEGFR2. A luciferase reporter assay confirmed that these miRNAs (16-5p/322-5p/497-5p) directly bind to VEGFR2. These data indicated that VEGFR2 is the target gene of these miRNAs (16-5p/322-5p/497-5p); moreover, exosomes from the plasma of Py-infected mice inhibited angiogenesis through these miRNAs (16-5p/17-5p/322-5p/497-5p). The impact of these miRNAs on angiogenesis was examined using tube formation assays. The miRNA 16-5p/17-5p/322-5p/497-5p decreased MS1 tube formation. However, the tube formation abilities of MS1 cells co-cultured with the miRNA 16-5p/17-5p/322-5p/497-5p were increased after transfection with specific miRNA inhibitors. Therefore, our results indicated that the exosomes from the plasma of Py-infected mice suppressed angiogenesis through overexpression of the miRNA 16-5p/17-5p/322-5p/497-5p.

In summary, we report that exosomes from *Plasmodium*-infected hosts inhibited VEGFR2 expression and tube formation in endothelial cells. Using intra-tumoral injection, exosomes from *Plasmodium*-infected hosts suppressed angiogenesis *in vivo*. This study provides the first evidence of a relationship between *Plasmodium* infection and tumor angiogenesis. This study advances our understanding of the actions of exosomes from plasma of malaria-infected hosts with regard to angiogenesis and provides further insight into potential exosome-based therapeutics.

## Materials and methods

### Ethics statement

In accordance with the ethics of our Institute, approval was obtained for the facilities used for all our animal experiments. The department of science and Technology of Guangdong province issued the approval (SYXK 2005-0063). Every experimental procedure used in this study was in compliance with the laid down rules governing animal use and handling in our Institute (Welfare Assurance #5748-01).

### Source of cells, parasites and mice

Murine LLC cells and mouse pancreatic islet endothelial cell (MS1) were obtained from ATCC. The nonlethal *P. yoelii* 17XNL (Py) strain was obtained from the malaria Research and Reference Reagent Resource center (MR4). Cell culture medium used in this experiment was Dulbecco’s modified eagle’s medium supplemented with penicillin (80 U/ml), streptomycin (100 U/ml) and 10% fetal bovine serum in a humidified atmosphere of 5% CO_2_ at 37 °C. Six-week-old female C57BL/6 mice were obtained from Slac Laboratory Animal Company (Shanghai, China) and raised in the animal facility.

### Animal experimental model

The weight of the animals were taken and then randomly divided into four groups (*n*=15 per group). There were no significant differences in weight between the groups. Mice were subcutaneously (s.c.) injected with 5 × 10^5^ LLC cells. Tumor-bearing mice injected with 5 × 10^5^ Py-infected red blood cells (RBCs) served as the Py+LLC group. Mice injected with only 5 × 10^5^ Py-infected RBCs served as the Py group. Tumor-bearing mice injected with non-infected erythrocytes were designated as the LLC group while mice with neither LLC nor Py injection were used as the naïve group.

### Exosome isolation

Whole blood isolated from mice was centrifuged at 3000 *g* for 15 min to remove cells and cell debris. Exosomes were isolated by ultracentrifugation or Exoquick-TC (SBI System Biosciences cat. No. EXOQ5A) methods according to the standard procedures or the manufacturer’s instruction (Supplementary Figure S4). Precipitation of exosomes from the supernatant (plasma) was in accordance with the manufacturer’s protocol. In brief, samples were incubated at room temperature for 30 min and then centrifuged at 1500 *g* for 30 min. Exosomes-rich fraction was washed twice with PBS and either stored at −80 °C or directly used in additional experiments.

### Characterization of exosome numbers, size and morphology

Measurement and analysis of exosomes by Nanosight using approximately 1000 μl appropriately diluted in PBS (1000 times) exosomes preparations was performed on a NanoSight ns300 Malvern (Worcester, UK). Individual videos of 60 s for each sample were acquired using the maximum camera gain and analyzed by the NanoSight particle tracking software to determine particles density and size. Exosomes were fixed in 2% paraformaldehyde at 4 °C and analyzed using Electron microscope. Exosomes were then deposited onto formvar grid for 20 min. The grids were then fixed (1% glutaraldehyde for 5 min), washed with distilled water and negatively stained with 4% uranyl acetate and 2% methyl cellulose for 5 min. The grids were then thoroughly dried and subsequently observed by transmission electron microscope (Tecnai G2 spirit). The diameters of the exosomes were quantified from the obtained micrographs (Supplementary Figure S5). Analysis for the presence of exosomes special markers CD63, CD9, CD81 and Hsp70 antibodies (System Biosciences catalog no. EXOAB-KIT-1) was carried out using western blot.

### *In vivo* and *in vitro* exosome treatment

Animals were weighed at treatment and divided to five groups (PBS, Naïve ex, LLC ex, Py ex, and Py+LLC ex) randomly (*n*=7 in each group). *In vivo*, mice were subcutaneously injected with 5 × 10^5^ LLC cells. Seven days later, 50 μg of the exosomes isolated from the different groups was injected into each animal via intra-tumoral injection once every 2 days for 8 days. On day 19, the mice were killed for tumor detection. In order to calculate tumor volume, tumor diameter was measured daily using digital calipers. Calculations of tumor volume was performed using the formula *V*=(*ab*2)/2, where *a* stands for the long axis and *b* represent the short axis.

*In vitro*, MS1 cells were seeded into a six-well plate at a density of 2 × 10^5^ cells, and 0.2 μg/μl exosomes from the different groups was added to the MS1 culture medium. After 24 h, cells were collected for further investigation.

### Exosomes uptake assays

Approximately one billion exosomes were labeled by incubation for 30 min with 1.0 mmol/l Dir Genecopoeia (Rockville, MD, USA) followed by re-precipitation to remove the excess Dir. On a 35 mm glass bottom μ-Dishes Ibidi (Planegg, Martinsried, Germany) 2 × 10^4^ cells were incubated with 1 × 10^9^ exosomes at 6 h and 2 mg/ml Hoechst 33342 Sigma (Sigma Chemical Co., St Louis, MO, USA) for 15 min before analysis. Excess exosomes were removed by washing with culture medium. Confocal images were collected with Multiphoton Laser Confocal Microscope (Zeiss 710 NLO) equipped with a × 63 oil-immersion objective. For western blot analysis, exosome were added in MS1 culture medium at 24 h. Excess exosomes were removed by PBS. MS1 cells were lysed in buffer supplemented with protease inhibitor cocktail. Antibodies were used in accordance to the manufacturer’s instructions: CD235a (abcom, catalog no. ab35760).

### RNA-Seq

The isolation of total RNA from samples was performed using TRIzol reagent Invitrogen (Carlsbad, VI, USA). The obtained total RNA was reverse transcribed to cDNA. RNA sequence was then carried out by Anoroad company (Beijing, China).

### MS1 tube formation

A 96-well plate coated with 50 μl of Matrigel BD Biosciences (Sparks, MD, USA) per well was solidified at 37 °C for 30 min. MS1 cells (2 × 10^4^/well) were seeded onto the 96-well plate for 8 h in presence of either 0.2 μg/μl exosomes or 50 nM miRNA mimics. Enclosed capillary networks of tubes were examined and imaged using a microscope (Nikon). The total tubes length was analyzed by ImageJ software.

### Transwell migration of endothelial cells

For the transwell migration assay, 1 × 10^5^ MS1 cells were seeded into the upper chamber (with 8.0 μm pores, BD Biosciences) in serum-free RPMI 1640. Complete culture medium and 0.2 μg/μl exosomes from different groups were loaded in the lower compartment. The lower chamber was seeded with or without LLC as positive and negative controls, respectively. After 20 h, the migrated or invaded cells were fixed with 100% methanol, stained with a hematoxylin solution (Sigma), and counted in five randomly selected optical fields.

### Scratch-wound assay

MS1 cells (5 × 10^5^/well) were seeded into a six-well plate and then the confluent monolayer was scratched with a yellow tip to create a wound. The monolayer was rinsed with PBS three times, the cells were co-cultured with 0.2 μg/μl exosomes from different groups for 24 h. Images were acquired immediately (*t*=0 h) or 24 h later (*t*=24 h) under a microscope (Olympus, IX51, Japan).

### Cell transfection

miRNA (16-5p/17-5p/322-5p/497-5p) mimics, mimic controls, miRNA (16-5p/17-5p/322-5p/497-5p) inhibitors and inhibitor controls were purchased from RiboBio Co., Ltd. (Guangzhou, China). Lipofectamine 3000 reagent (Invitrogen) was used to transiently transfect the miRNAs (50 nM) into target cells as described in the product manual.

### Quantitative polymerase chain reaction (qPCR)

The isolation of total RNA from MS1 cells was performed using TRIzol Reagent (Invitrogen). The obtained total RNA was reversely transcribed to cDNA using Primescript RT reagent Kit TaKaRa (Kusatsu, Shiga, Japan). To evaluate the mRNA levels of a number of genes, qRT–PCR was performed on a CFX96 qRT–PCR System using SYBR Green qRT–PCR master mix (Promega, Madison, WI, USA). GAPDH (glyceraldehyde-3-phosphate dehydrogenase) was used as the internal control. All of the samples were normalized to internal controls, and fold changes were calculated based on relative quantification (2^−△△^Ct).

### Immunoblotting

Cells were lysed, total protein concentration was measured by bicinchoninic acid method. Thirty micrograms of protein was electrophoresed on 8% SDS–PAGE gel and transferred to a PVDF (polyvinylidene fluoride) membrane (Millipore, Billerica, MA, USA). After blocking the membrane, it was incubated with primary antibody (rabbit anti-VEGFR2 and anti-phospho-VEGFR2 monoclonal antibodies, 1:1000; catalog no.8696 S, Cell Signaling Technology, Danvers, MA, USA) at 4 °C overnight. The membrane was washed with Tris-buffered saline containing 0.1% Tween-20 (TBST) and incubated with horseradish peroxidase-conjugated secondary antibody. The protein bands were detected by chemiluminescence (Sigma) and visualized using BioImaging Systems. Protein expression was normalized to β-actin levels.

### Subcloning the 3′-UTRs VEGFR2 into vector

VEGFR2 3′-UTR including miR-16-5p/322-5p/497-5p target sequence was subcloned into the psicheck2**-**vector RiboBio (Guangzhou, Guangdong, China) using the touchdown PCR method and the following primers:

3′-UTR-VEGFR2 forward, 5′-TCGAGTGAAATAGCAAACCCGAGTTTCTTCCTCTGCTGCTGGCCATTTCCTAAACAGC-3′

3′-UTR-VEGFR2 reverse,

5′-GGCCGCTGTTTAGGAAATGGCCAGCAGCAGAGGAAGAAACTCGGGTTTGCTATTTCAC-3′

3′-UTR-VEGFR2 mutant forward,

5′-TCGAGTGAAATAGCAAACCCGAGTTTCTTCCTCTCTCGTCGGCCATTTCCTAAACAGC-3′

3′-UTR-VEGFR2 mutant reverse,

5′-GGCCGCTGTTTAGGAAATGGCCGACGAGAGAGGAAGAAACTCGGGTTTGCTATTTCAC-3′.

Phase 1 included denaturation at 95 °C for 5 min followed by a room temperature incubation for 1 h. In phase 2, the obtained fragments and the psicheck2**-**vector were ligated by T4 ligase. The wild-type and mutant sequences were confirmed by DNA sequencing.

### Dual luciferase reporter assay

Cells were incubated with Lipofectamine 3000 transfection reagent Life Technology (Waltham, MA, USA) and the psicheck2 vectors for 24 h and then subjected to a luciferase reporter assay using the Dual Luciferase Assay System (Promega, Madison, WI, USA) according to the manufacturer’s instructions.

### Immunohistochemistry (IHC)

Tumors were fixed in 4% neutral paraformaldehyde and embedded in paraffin. The paraffin-embedded sections were subjected to high pressure for 2 min for antigenic retrieval. Identification of endothelial cells was performed by immunostaining with anti-CD63, anti-CD235a and anti-CD31 monoclonal antibody Abcam (Cambridge, MA, USA). Briefly, the sections were incubated with a 1:100 antibody dilution at 4 °C overnight followed by exposure to the secondary antibody, and the signals were detected by staining the sections with 3,3′-diaminobenzidine and counterstaining with hematoxylin. To evaluate CD31 expression, semiquantitative image analysis of the immunohistochemical section was used to measure the integrated optical density using Image Pro Plus software.

### Statistical analysis

Data are shown as the mean±s.e.m. *P-*values were calculated using either one-way ANOVA or Student’s *t*-test. *P*<0.05 or less was considered statistically significant.

## Figures and Tables

**Table 1:** Gene ontology (GO) analysis of the up- and downregulated genes associated with cell proliferation, angiogenesis, migration and vessels development

*GO ID*	*Biological process (GO description)*	P*-value*	*Count*
GO:0008283	Cell proliferation	8.80E-07	37
GO:0042127	Regulation of cell proliferation	1.10E-06	32
GO:0043542	Endothelial cell migration	6.10E-05	8
GO:0045765	Regulation of angiogenesis	9.30E-05	9
GO:1901342	Regulation of vasculature development	0.00014	9
GO:0001568	Blood vessel development	0.0002	16
GO:0048514	Blood vessel morphogenesis	0.0002	15
GO:0001525	Angiogenesis	0.00024	13
GO:0035239	Tube morphogenesis	0.00028	12
GO:0002040	Sprouting angiogenesis	0.00032	5
GO:0001944	Vasculature development	0.00036	16
GO:0030336	Negative regulation of cell migration	0.00147	7
GO:0010574	Regulation of vascular endothelial growth factor production	0.00284	3
GO:0010594	Regulation of endothelial cell migration	0.00284	5
GO:0010573	Vascular endothelial growth factor production	0.00324	3
GO:0016525	Negative regulation of angiogenesis	0.00355	4
GO:0010596	Negative regulation of endothelial cell migration	0.00367	3
GO:0035150	Regulation of tube size	0.00377	5
GO:0050880	Regulation of blood vessel size	0.00377	5
GO:0043534	Blood vessel endothelial cell migration	0.00474	4

Comparison between exosomes from infected mice and exosomes from non-infected mice co-cultured with MS1 cells. We identified and classified mRNAs showing a greater than twofold effect in expression with *P-*values below 0.05 using GO categories.
