# Supplementary material for: Exosomes from Plasmodium-infected hosts inhibit tumor angiogenesis in a murine Lewis lung cancer model
Source: Oncogenesis. 2017 Jun 26;6(6):e351–. doi: 10.1038/oncsis.2017.52 (PMC5519199; doi:10.1038/oncsis.2017.52)
Supplement: Supplementary Figure legend [file oncsis201752x1.docx]

Supplementary S1. Haematoxylin & eosin staining detected neovascularization in tumor tissues at 19 days. Scale bar 50μm.

Supplementary S2. Anti-CD63 and anti-CD235a validated the exosomes in tumor tissue by Immunohistochemical. Scale bar 50μm

Supplementary S3. a. Schematic of VEGF-α/VEGFR2 signal pathway. b. Western blotting analysis of proteins of VEGF/VEGFR2 pathway expression in MS1 cells co-culture with exosomes. Ki8751 was used as a control. c. Western blotting analysis of DLL4 expression in MS1 cells co-culture with exosomes. d. Endothelial cell tube formation assay showed interference of network assembly of MS1 cells on pre-solidified Matrigel in medium containing Ki8751. Scale bar 500μm. (**p*<0.05, compared with control without Ki8751 group)

Supplementary S4. Particle numbers and size of ExoQuick (EQ) and ultracentrifugation (UC) isolated exosomes distributions obtained by nanosight. Left: Exosomes numbers from two methods isolated. Right: size of EQ and UC isolated exosomes (**p*<0.05). The results clearly indicated that exosomes from both methods are similar in terms of size. This shows that what we used was clearly exosomes. Furthermore, one of the advantages of using isolation kit is high exosomes yield.

Supplementary S5. TEM graphs from exosomes extracted with EQ method (left) and UC (right).

Supplementary S6. Effect of exosomes on cells proliferation. a. Number of LLC co-culture with different exosomes at 24 hours. b. Number of MS1 co-culture with different exosomes at 24 hours (**p*<0.05). c. CCK8 assay on proliferation of MS1 co-culture with different exosomes at 24 hours (**p*<0.05).
