# Supplementary figures and images for: Exosomes from Plasmodium-infected hosts inhibit tumor angiogenesis in a murine Lewis lung cancer model
Source: Oncogenesis. 2017 Jun 26;6(6):e351–. doi: 10.1038/oncsis.2017.52 (PMC5519199; doi:10.1038/oncsis.2017.52)

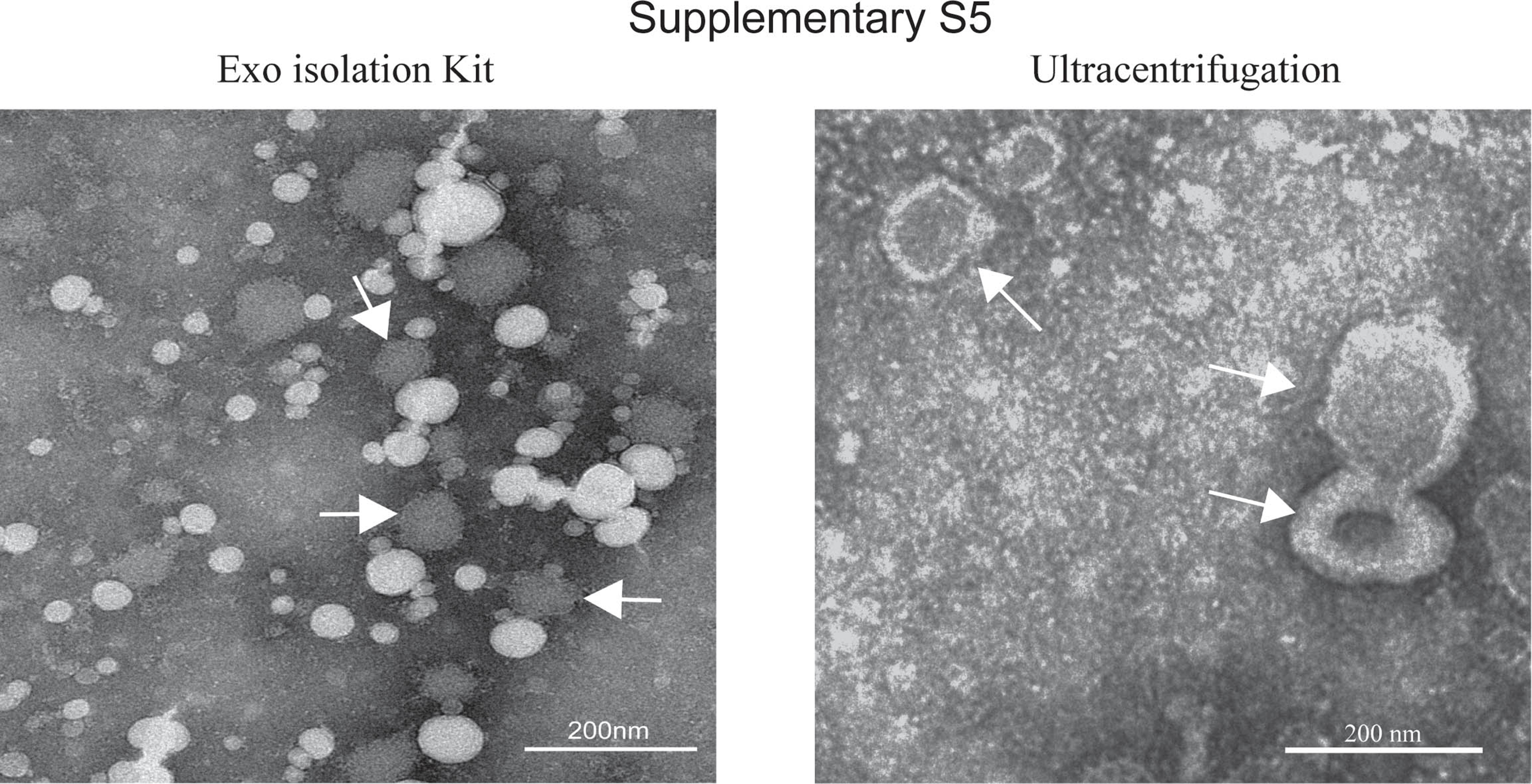

Supplement: Supplementary Figure S5 [file oncsis201752x6.tif]
